# Risk for mental illness following exposure to violence and threats among newly arrived refugees

**DOI:** 10.1186/s13104-022-06239-1

**Published:** 2022-12-09

**Authors:** Elisabeth Mangrio, Slobodan Zdravkovic, Anna-Karin Ivert

**Affiliations:** 1 Department of Care Science, Faculty of Health and Society, Malmö University, Malmö, Sweden; 2 Diversity and Welfare (MIM), Malmö Institute for Studies of Migration, Malmö University, Malmö, Sweden; 3 Department of Criminology, Faculty of Health and Society, Malmö University, Jan Waldenströms Gata 25, 205 06 Malmö, Sweden

**Keywords:** Mental illness, Newly arrived refugees, Threats, Violence, Quantitative research

## Abstract

**Objective:**

There is an association between pre-migration exposure to threats and violence, and the risk for mental illness among newly arrived refugees (NAR). The aim of this study is therefore to investigate the effect of pre-migration violent and threatening experiences on the mental health of NAR in Sweden. The participants were recruited between February 2015 and February 2016, undergoing the naturalisation process in Sweden. In total, 681 questionnaires were returned (response rate of 39.5%).

**Results:**

The results showed that almost 50% of the sample were at risk for mental illness. Analysis of pre-migration exposure to violence or threats, and risk for mental illness, showed a significant odds ratio for violence as well as for threats. Analysing men and women separately resulted in a significant odds ratio for women for pre-migration threats. For men, pre-migration violence and threats were significantly associated with the risk for mental illness.

The host society receiving NAR must screen for mental illness and be prepared to provide support and care for refugees who were exposed to violence or threats, and who are subsequently at risk for mental illness. This must be considered in order to improve health and subsequently the social integration of refugees.

**Supplementary Information:**

The online version contains supplementary material available at 10.1186/s13104-022-06239-1.

## Introduction

In 2015, the number of refugees entering Europe increased significantly [1] and that year, more than 160,000 persons applied for asylum in Sweden [2]. The migration process often entails being separated from family members, and thus lacking a support network [3, 4], while the process also increases the odds for exposure to violence [5]. After the arrival in the host country, the refugees face challenges finding safe housing, which in turn could increase the risk for exposure to violence [6, 7]. We are also aware that women are exposed to an increased risk of violence, including forced sexual relations, sexual violence from partners, or through trafficking [8].

Prevalence of depression and anxiety among Newly arrived refugees (NAR) tends to be higher than in host populations [9] and poor socioeconomic conditions, including unemployment and isolation, are associated with increased rates of depression among refugees [10]. Health, both physical and mental, is important for NAR, in order to be able to participate in the establishment process [11]. Therefore, there is a need to examine risk factors among NAR that are associated with mental health problems. The objective of the present study is to investigate the effect of pre-migration experiences of violence and threats on mental health among a sample of NAR in Sweden. Gender differences will also be investigated in order to determine whether experiences of violence and/or threats affect females and males differently.

## Main text

### Materials and methods

Approximately 1,700 questionnaires were distributed to NAR who spoke Dari and Arabic (from Syria, Iraq and Afghanistan), and who participated in the mandatory 2 year naturalisation process in Scania, Sweden. At the time of the survey, the NAR had been granted refugee status and received either temporary or permanent residency permits. The participants were recruited by inviting all eligible adult NAR between February 2015 and February 2016 [12]. In total, 681 questionnaires were returned, resulting in a response rate of 39.5%. Sixteen individuals with missing gender data were excluded, resulting in a final sample of 665 respondents. Data collection was assessed by a self-administered questionnaire that included questions about health, sleep, level of education, well-being, accommodation type, and social relations. The questions were translated by authorised translators, and validated by civic and health communicators. A pilot study among civic and health communicators that themselves were newly arrived in Sweden as refugees, was conducted prior to the study in order to validate comprehension of the questionnaire. After the pilot study, minor adjustments were made.

Prior to the data collection, the NAR got oral and written information about the study and the questionnaires were distributed along with the civic and society information and the NAR received the questionary and were able to ask the civic and society communicators with help if questions were difficult to understand. Then the informants posted the questionnaires in a pre-packed envelope. There was no way to measure what level of health literacy the informants had at the time of the survey.

#### Variables

##### Dependent variable

In this study, the General Health Questionnaire (GHQ-12) [13] was used to examine the risk of mental illness. The instrument is a 12-item questionnaire with a four-point Likert scale measuring a person’s well-being, including mainly depressive symptoms, worry, sleep and cognitive functioning. A score sum of  ≥ 3 according to Goldberg’s [13] original recommendation was used.

##### Independent variables

*Pre-migration exposure to violence* was derived from the following question: Have you been exposed to physical violence during the last 12 months before you came to Sweden that were so serious that you got afraid? Answers: yes, no.

*Post-migration exposure to violence* was derived from the following question: Have you been exposed to physical violence during the last 12 months being in Sweden? Answers: yes, no.

*Pre-migration exposure to threats* was derived from the following question: Have you been exposed to threats of violence during the last 12 months before you came to Sweden that were so serious that you got afraid?

*Post-migration exposure to threats* was derived from the following question: Have you been exposed to threats of violence during the last 12 months being in Sweden? Answers: yes, no.

*Gender* was divided into male or female. Being male was used as reference category in the analysis.

*Age* was measured as a continuous variable.

*Marital status* was derived from the question: What is your marital status? The answers were divided between married/cohabiting (reference category), not married, divorced and widow/widower. Due to small numbers, the latter three categories were collapsed in to one category.

*Educational level* was based on years of schooling, divided into low educational level (9 years or less), medium educational level (10–12 years of school) and high educational level (more than 12 years). Low educational level was used as reference.

#### Statistical analyses

Descriptive analyses were performed as frequencies and percentages for qualitative variables, and as mean and standard deviation for quantitative variables. Chi-square and t-test were used for testing differences between groups. The association between the outcome and exposure variables was analysed by logistic regression in terms of odds ratios and 95% confidence intervals.

### Results

In total, 665 NAR were included in the analysis of this paper. Of these 665 NAR, 47.2% were at risk for mental illness (45.3% women and 48% men). 19.4% experienced pre-migration exposure to violence. There was a significant difference in pre-migration violence between men and women (10.8% women and 23.2% men). 58.6% experienced pre-migration exposure to threats, and here too there was a significant difference between men and women (48.5% women and 63.1% men). 1–7% experienced post-migration exposure to violence (2.2% women and 1.5% men), and here there was no significant difference between the genders. 5.7% experienced post-migration exposure to threats (5.5% women and 5.8% men) and there was no significant difference between men and women. Results are shown as Additional file 1.

When analysing pre-migration exposure to violence or threats, in relation to the risk for mental illness (Table 1), there was a significant crude odds ratio (OR) of 1.99 (95% CI 1.26–3.15, p < 0.01) for violence as well as for threats, at 1.76 (1.21–2.54, p < 0.01). When adjusting for gender, age and educational level, both OR remained significant at 1.99 (1.25–3.19, p < 0.01) and 1.82 (1.25–2.66, p < 0.01). Analysing men and women separately resulted in significant OR for women for pre-migration threats both as crude OR, 2.46 (1.25–4.82, p < 0.01), and as adjusted, 2.82 (1.40–5.70, p < 0.01), see Table 2. For men, pre-migration violence was significant both as crude OR, 2.31 (1.38–3.85, p < 0.01), and as adjusted OR, 2.26 (1.35–3.80, < 0.01). The OR of pre-migration threats for men, after adjusting for age, education, and marital status, was significant at 1.62 (1.03–2.56, p < 0.05), see Table 3.

**Table 1:** Logistic regression predicting mental ill-health

Variables	Model 1		Model 2	
	OR	95% CI	OR	95% CI
Pre-migration exposure to violence	1.991**	1.26–3.15	1.995**	1.25–3.19
Pre-migration exposure to threats	1.757**	1.21–2.54	1.822**	1.25–2.66
Gender				
Male			1	
Female			0.958^ ns^	0.65–1.42
Age			0.980^ ns^	0.96–1.0
Marital status				
Married or cohabiting			1	
Other			0.812^ ns^	0.53–1.24
Education level				
> 9 years			1	
10–12 years			0.898^ ns^	0.54–1.49
12 + years of education			1.019^ ns^	0.66–1.57

*OR* odds ratios and *CI* 95% confidence intervals

^*^p < 0.05; ** < 0.01, ***p < 0.001

**Table 2:** Logistic regression predicting mental ill-health in women

Variables	Model 1		Model 2	
	OR	95% CI	OR	95% CI
Pre-migration exposure to violence	0.947^ ns^	0.31–2.92	0.835^ ns^	0.25–2.80
Pre-migration exposure to threats	2.458 ^**^	1.25–4.82	2.821^**^	1.40–5.70
Age			0.980^ ns^	0.95–1.01
Marital status				
Married or cohabiting			1	
Other			1.163^ ns^	0.54–2.52
Education level				
> 9 years			1	
10–12 years			0.406^ ns^	0.16–1.04
12 + years			0.501^ ns^	0.22–1.16

*OR* odds ratios and *CI* 95% confidence intervals

^*^p < 0.05; ** < 0.01, ***p < 0.001

**Table 3:** Logistic regression predicting mental ill-health in men

Variables	Model 1		Model 2	
	OR	95% CI	OR	95% CI
Pre-migration exposure to violence	2.307^**^	1.38–3.85	2.255^**^	1.35–3.80
Pre-migration exposure to threats	1.538^ ns^	0.98–2.41	1.619^*^	1.03–2.56
Age			0.976^ ns^	0.95–1.0
Marital status				
Married or cohabiting			1	
Other			0.704^ ns^	0.41–1.20
Education level				
> 9 years			1	
10–12 years			1.229^ ns^	0.67–2.26
12 + years			1.301^ ns^	0.78–2.17

*OR* odds ratios and *CI* 95% confidence intervals

^*^p < 0.05; ** < 0.01, ***p < 0.001

### Discussion

The results in the current study showed that almost 50% of the NAR were at risk for mental illness. Pre-migration exposure to violence or threats is associated with the risk for mental illness even after adjustments for confounders. After analysing men and women separately, it resulted in significant risk for women for pre-migration threats. For men, pre-migration violence was significant both as crude and as adjusted. Pre-migration threats was borderline significant for men after adjustments were made.

This result is in line with an earlier study, which showed an association between pre-migration exposure to violence and increased risk for mental health problems [14]. A newly-published systematic review confirms an association between pre-migration exposure to violence and mental health problems among resettled refugees [15].

In the current study, very few reported post-migration exposure to violence or threats, which may be due to underreporting. The pre- and post-exposure to threats and violence could as well be related to the number of children in the household as well as how long and in what way the NAR had to travel during the migration travel. But unfortunately, we do not have those questions in this data analyses which could be seen as a limitation. Marital status and educational level were used as confounders in the logistic regression, but no significant results were found. It could have been interesting to further on understand the association between exposure to threats and violence and marital status and educational level but has to be a focus for another study in the future.

Because of the risk that pre-exposure to threats and violence could have on the risk for mental ill-health among NAR in Sweden, it’s important that healthcare and social workers must acknowledge and support refugees during resettlement, since we know that some refugees may experience mental health risk factors before settling in the host country, and resettlement may entail additional psychological burdens which could worsen the mental health of these people [10]. We also know, from earlier research within MILSA, that NAR face challenges mentally after arrival in Sweden [16]. This research suggests that health- and social workers encounter NAR with the knowledge that the risk could be increased that they have been exposed to threats or violence before the arrival here and that they keep their eyes open for seeing signs that could indicate that such a risk is there.

### Conclusions

Pre-migration exposure to violence or threats increased the risk for mental illness among NAR in the county of Scania in Sweden. This indicates that there is a need to identify individuals with experiences of victimisation, but also the need to acknowledge and explore gender differences in the association between victimisation and mental illness. The host society receiving NAR needs to be prepared to support and care for these refugees in order to improve their health and therefore integration, since integration is closely linked to health among refugees. In order to be more prepared as a society in Sweden, further qualitative studies among both professionals working with NAR and as well among NAR, in order to illuminate the association between exposure to threats and risk for mental illness, even more.

### Limitations

The response rate in the current study was 39.5%, which could be considered low, and may have an effect on the generalisability of the results. However, the response rate is similar to or higher than comparable studies with the same field of research [17, 18].

The survey’s anonymity made it difficult to further analyse dropout. However, an approximate dropout analysis was performed by comparing the characteristics of the study participants with statistics from Sweden’s Division of Labour. This comparison suggested that people with higher levels of education could be overrepresented in the present study [19]. GHQ-12 was used for assessing risk for mental illness, and this instrument is well validated for assessing risk factors for mental illness [13, 20]. However, to the best of our knowledge, GHQ-12 is not especially adapted for studies focusing on refugees, but has been used in other studies focusing on refugees [21, 22].

Furthermore, conducting a cross-sectional study could be seen as a limitation, because it looks for correlations, and is limited in its ability to explain the reasons behind correlations. The NAR were asked to participate when they had received the residence permit to stay and could have been in Sweden between 1–2 years approximately. It could be discussed how well the informants could recall situations where they had been exposed to pre-migration threats or violence and could be seen as a limitation. However, we believe that there could be a quite underreporting due to sensitive and shameful feelings around the question. It could have been interesting to divide the NAR according to origin of birth and language, however, mostly NAR were Arabic speaking and came from Syria and very few were Dari speaking or came from Iraq or Afghanistan [19].

## Supplementary Information

**Additional file 1. **Descriptive statistics of study variables. Percentages or means. Individuals with missing information on gender (n=16) were excluded.

## Data Availability

Data cannot be shared due to requirements for the ethical approval of the study.
