# Supplementary material for: Risk for mental illness following exposure to violence and threats among newly arrived refugees
Source: BMC Res Notes. 2022 Dec 9;15:361. doi: 10.1186/s13104-022-06239-1 (PMC9733151; doi:10.1186/s13104-022-06239-1)
Supplement: Supplementary file 1 — Additional file 1. Descriptive statistics of study variables. Percentages or means. Individuals with missing information on gender (n=16) were excluded. [file 13104_2022_6239_MOESM1_ESM.docx]

| Supplementary material: Descriptive statistics of study variables. Percentages or means. Individuals with missing information on gender (n=16) were excluded. | | | | |
| --- | --- | --- | --- | --- |
| Variables | Total population (N=665) | Females (n=204) | Males (n=461) | Between gender difference |
| Variables |  |  |  | P-value |
| Psychological distress | 47 | 45.3 | 48 | NS |
| Pre-migration exposure to violence | 19.4 | 10,8 | 23.2 | < .001 |
| Pre-migration exposure to threats | 58,6 | 48.5 | 63.1 | < .001 |
| Post-migration exposure to violence | 1.7 | 2.2 | 1.5 | NS |
| Post-migration exposure to threats | 5.7 | 5.5 | 5.8 | NS |
| **Age** (mean) | 36.1 | 36.5 | 35.9 | NS |
| **Marital status** |  |  |  | .004 |
| Married or cohabiting | 65.1 | 73 | 61.5 |  |
| Other | 30.4 | 17.2 | 36.3 |  |
| **Education level** |  |  |  | NS |
| >9 years | 25.7 | 26.5 | 25.3 |  |
| 10–12 years | 23.7 | 26 | 22.7 |  |
| More than 12 years of education | 50.6 | 47.4 | 52 |  |
